# A highly CMOS compatible hafnia-based ferroelectric diode

**DOI:** 10.1038/s41467-020-15159-2

**Published:** 2020-03-13

**Authors:** Qing Luo, Yan Cheng, Jianguo Yang, Rongrong Cao, Haili Ma, Yang Yang, Rong Huang, Wei Wei, Yonghui Zheng, Tiancheng Gong, Jie Yu, Xiaoxin Xu, Peng Yuan, Xiaoyan Li, Lu Tai, Haoran Yu, Dashan Shang, Qi Liu, Bing Yu, Qiwei Ren, Hangbing Lv, Ming Liu

**Affiliations:** 1 Key Laboratory of Microelectronics Devices and Integrated Technology, Institute of Microelectronics of the Chinese Academy of Sciences, No. 3 Beitucheng West Road, Chaoyang District, Beijing, 100029 China; 2 Key Laboratory of Polar Materials and Devices (MOE), Department of Electronics, East China Normal University, 500 Dongchuan Road, Shanghai, 200241 China; 3 Xi’an UniIC Semiconductors Co., Ltd., 38 Gaoxin 6th Rd, High-Tech Industrial Development Zone, Xi’an, 710075 China

**Keywords:** Electrical and electronic engineering, Electronic devices, Electronic properties and materials

## Abstract

Memory devices with high speed and high density are highly desired to address the ‘memory wall’ issue. Here we demonstrated a highly scalable, three-dimensional stackable ferroelectric diode, with its rectifying polarity modulated by the polarization reversal of Hf_0.5_Zr_0.5_O_2_ films. By visualizing the hafnium/zirconium lattice order and oxygen lattice order with atomic-resolution spherical aberration-corrected STEM, we revealed the correlation between the spontaneous polarization of Hf_0.5_Zr_0.5_O_2_ film and the displacement of oxygen atom, thus unambiguously identified the non-centrosymmetric Pca2_1_ orthorhombic phase in Hf_0.5_Zr_0.5_O_2_ film. We further implemented this ferroelectric diode in an 8 layers 3D array. Operation speed as high as 20 ns and robust endurance of more than 10^9^ were demonstrated. The built-in nonlinearity of more than 100 guarantees its self-selective property that eliminates the need for external selectors to suppress the leakage current in large array. This work opens up new opportunities for future memory hierarchy evolution.

## Abstract (web summary)

Designing reliable, scalable and high speed computing systems remains a challenge. Here, the authors identify noncentrosymmetric orthorhombic phase in HZO film and demonstrate a CMOS compatible 3D Vertical HZO-based ferroelectric diode array with self-selective property and 20 ns of speed operation.

## Introduction

Memory hierarchy composed of volatile and nonvolatile solid-state memories is the main building block of the computing system^1^. It could achieve an optimal trade-off between performance and density in different types of memory^2^. However, the frequent data transfer between different memories leads to the decreased bandwidth and degraded computing efficiency, especially in the case of massive data computing, resulting in the well-known “memory wall” issue. To effectively solve the memory wall problem, a desired way is to configure a memory with high speed and high-density features.

Among various types of nonvolatile memories, ferroelectric random-access memories (FeRAM), which achieve nonvolatility by switching and sensing the polarization state of a ferroelectric capacitor, have been thought of as an excellent memory solution due to its outstanding features of low power, high speed, high endurance, and good retention. However, the scaling limitation of perovskite ferroelectric materials has hindered their widespread applications^3^. The development of FeRAM in semiconductor industry has been stopped at 130-nm node for a long time^4,5^. The discovery of ferroelectricity in doped HfO_2_ has renewed the interest in FeRAM by offering a possible solution to bridge the scaling gap between perovskite ferroelectric materials and complementary metal–oxide–semiconductor (CMOS) technology^4,6^.

Up to now, ferroelectric HfO_2_-based memories in the forms of one access transistor–one ferroelectric capacitor (1T1C)^5,7^ and ferroelectric transistor (FeFET) structures have been demonstrated, with excellent scalability down to 10-nm node^8^. As shown in Supplementary Table 1, the 1T1C FeRAM requires a large capacitor area (“footprint”) and undergoes destructive readout. FeFET can realize non-distructive read and 3D vertical stack^9–11^. However, it cannot be used as a random-access memory. On the other hand, two terminal devices, such as the ferroelectric tunneling junction (FTJ) and Fe diode, have the potential to achieve high- density crossbar array^12–15^. The FTJ device takes the advantage of a ferroelectric as the barrier material, and has a giant tunnel electroresistance (TER) effect by switching the ferroelectric polarization, which was generally formulated by quantum mechanical electron-tunneling mechanism. In principle, both the on and off states of FTJ obey linear or quasi-linear *I*–*V* relationship, which makes it need extra selector device to diminish the sneaking current in the crossbar array. In contrast, the ferroelectric diode (Fe diode), with the working principle governed by Schottky barrier modulation as polarization reversal, has the potential to gain inherent nonlinearity and realize selector-free cross-point integration. The concept of Fe diode was first proposed in PbTiO_3_ perovskite thin films^16^ and later in bulk BiFeO_3_ single crystals^17^. The ultralow readout currents in the orders of 20 mA/cm^2^ and low on/off ratio limited their miniaturization. In 2011, Jiang et al. reported a Fe-diode current density up to 5.4 A/cm^2^ in Pt/BiFeO_3_/SrRuO_3_ thin-film capacitors by a plate-like growth mode^18^. However, it is still too low to be detected by a periphery circuit in chip. Meanwhile, the CMOS- incompatible film growth technique makes the current state of the art of Fe diode unable to be implemented in the 3D structure.

Here, we proposed a hafnia-based switchable Fe diode by spontaneous polarization reversal in the non-centrosymmetric Pca2_1_ orthorhombic phase. First, the non-centrosymmetric orthorhombic phase (Pca2_1_) in Hf_0.5_Zr_0.5_O_2_ (HZO) film was unambiguously identified by visualizing both the hafnium/zirconium lattice order and oxygen lattice order with atomic-resolution spherical aberration (Cs)-corrected scanning transmission electron microscopy (STEM). The displacement of oxygen atoms was ascribed as the root of the spontaneous polarization that occurred in the domain. Then, based on this promising ferroelectric material, we demonstrated a fully CMOS-compatible Fe diode with high readout current density (>200 A/cm^2^) and built-in nonlinearity (>100), which was further 3D integrated up to eight layers with vertical size down to 20 nm. High operation speed of 20 ns and good endurance properties were achieved, showing its promising prospect to achieve high speed and high density. This work opens up new opportunities for future memory hierarchy evolution in the computing system.

## Results

### Identification of the non-centrosymmetric Pca2_1_ phase

**Fig. 1:**
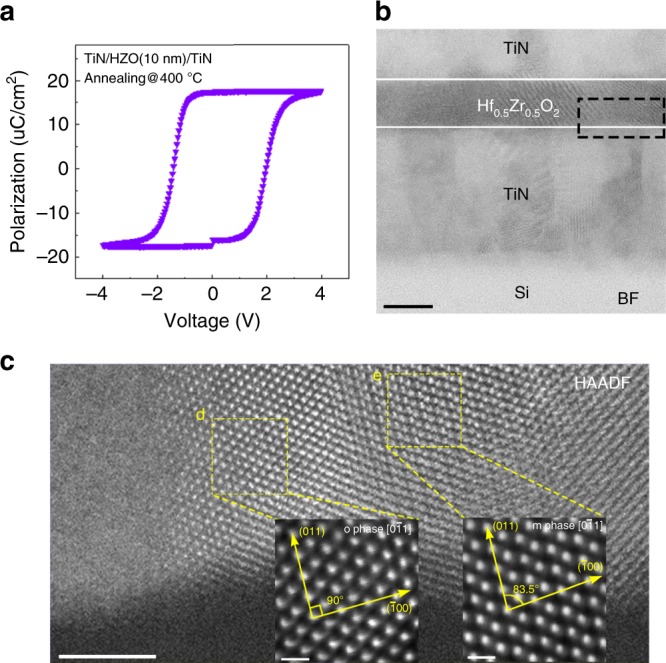
Planar metal–ferroelectric–metal (MFM) capacitors. **a** Typical P–V loop (2.5 kHz) of the Hf_0.5_Zr_0.5_O_2_ (HZO) film derived from positive-up negative-down (PUND) measurements. **b** STEM-BF image of the TiN/HZO/TiN stack. Scale bars, 10 nm. **c** STEM-HAADF image of the HZO thin film with two specific crystal grains, projected along o [$0\bar 11$] (left square) and m [$0\bar 11$] (right square) zone axes. Scale bars, 5 nm. Scale bar inset, 0.5 nm.

Metal–ferroelectric–metal (MFM) capacitors with HZO film (thickness of 10 nm) were fabricated and characterized to confirm its ferroelectricity. The detailed material deposition and device fabrication processes were described in the “Experimental” section and Supplementary Fig. 1. To elucidate the FE properties of TiN/HZO/TiN samples, the measurements were performed using the PUND (positive-up negative-down) technique^19^. Figure 1a is the typical hysteresis P–V loop of TiN/HZO/TiN device after 400 °C of rapid thermal annealing, showing a remnant polarization (Pr) of ~17 μC/cm^2^ and coercive field of −1.4 MV/cm and +1.9 MV/cm. Figure 1b depicts the bright-field (BF) STEM image of TiN/HZO/TiN stack, in which the polycrystalline nature of the HZO thin film as well as the boundaries with TiN metal electrodes are visible. In the close-up image of the HZO film (Fig. 1c), o- and m-phases can be identified in certain directions because the m-phase has a non-vertical β angle. Based on the apparent relative angle differences between the two corresponding lattice planes, orthorhombic [$0\bar 11$] (left square) and monoclinic [$0\bar 11$] grains (right square) were clearly identified, indicating the coexistence of the two phases in the HZO film.

It is generally accepted that the orthorhombic phase (o-phase) is the origin of ferroelectricity^6,20,21^. However, there are four different space groups in the o-phase, i.e., Pca2_1_, Pbcm, Pmn2_1_, and Pbca, among which, only the Pca2_1_ and Pmn2_1_ space groups are the polar phase. The Pmn2_1_ phase could be easily ruled out by the image of the hafnium sublattice. However, distinguishing between the Pbca, Pbcm, and Pca2_1_ space groups is quite challenging, which could only be realized by visualizing the tiny difference of oxygen sublattice.

Sang et al.^21^ reported the presence of the o-phase in Gd-doped HfO_2_ films by using the atomic-resolution STEM high-angle annular dark-field (HAADF) technique, in which the projected hafnium sublattices are in good agreement with the o-lattice. Further, in order to distinguish the different space groups of the o-phase, they showed the position-averaged convergent beam electron diffraction (PACBED^22^) patterns from a certain o-grain that lacks mirror symmetry, to imply the existence of non-centrosymmetric orthorhombic Pca2_1_ structure. However, there is no directly convincing evidence to distinguish the different o-space groups and elucidate the origin of the noncentrosymmetry of Pca2_1_. Only the oxygen displacement along the polarization direction is maximum; the visible shift in atomic scale has never been experimentally reported. Hence, solid evidences on short- range ordering of the oxygen sublattice and displacement in the local domain are essentially required.

**Fig. 2:**
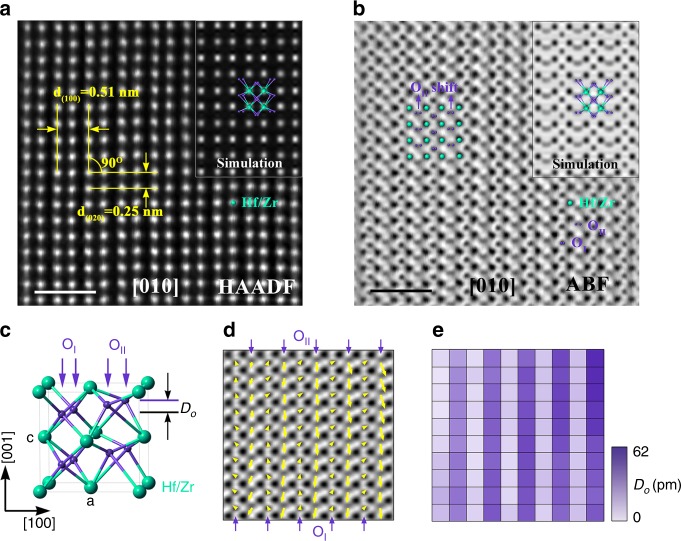
Atomic structure of HZO orthorhombic phase. **a** Scanning transmission electron microscopy high-angle annular dark-field (STEM-HAADF) image of the HZO grain projected along O [010] zone axes with the inset of simulated HAADF picture. Scale bar, 1 nm. **b** The corresponding scanning transmission electron microscopy high-angle annular dark-field annular bright-field (STEM-ABF) image of (**a**) with the inset of simulated ABF picture. Scale bar, 1 nm. **c** Schematic of the unit cell of HZO, where *D*_O_ denotes the relative displacement of O atoms. **d** O atomic displacement vector map from the area in (**b**). The yellow arrows indicate reversed O atom displacement (*D*_O_) vectors, which were consistent with the spontaneous polarization direction in (**b**). **e** Two-dimensional O atomic displacement map relative to the central positions of the four nearest heavy atoms, which corresponds to the yellow arrows in (**d**).

In order to elucidate the ferroelectric nature of the orthorhombic phase, the sublattice order of hafnium/zirconium and oxygen atoms was directly detected by using the atomic STEM-HAADF and ABF technique. For HZO lattice, only one direction can maximize the difference in oxygen atomic displacement to identify the different o-phase space groups. Here, [010] is the most suitable zone axis to project the atom columns. It can distinguish monoclinic, orthorhombic, and tetragonal phases, and it is also relatively easily to tell the differences in the oxygen atom arrangement, as shown in Supplementary Fig. 2. Figure 2a is a typical atomic STEM-HAADF image of the HZO o-phase, projected along the [010] zone axes, which fits well with the simulated result (inset) using the Pca2_1_ space group. In this HAADF image, only heavy atoms such as Hf and Zr can be identified, which have very similar arrangement^21^ in different space groups of the o-phase. Further, atomic-resolution STEM-ABF technique, which is sensitive to light elements, was used to inspect the information of oxygen atoms. Figure 2b is the corresponding ABF experimental image of the o-phase projected along the [010] zone axes. It includes the inset of the simulated result and the overlaid model using the o-phase with Pca2_1_ space group. The shift of oxygen atoms is indicated by purple arrows. The experimental and simulated results are completely consistent, intuitively confirming the existence of non-centrosymmetric Pca2_1_ structure in the hafnia ferroelectric material. In addition, Supplementary Fig. 3 gives the simulated ABF pictures of the o-phase with Pbcm and Pbca space groups, which are totally different from the experimental result presented in Fig. 2b. Figure 2c is the schematic of the HZO unit cell, in which, two kinds of oxygen (O_I_, O_II_) with different displacement behavior were denoted, and *D*_O_ is the shift distance of the oxygen atoms. Afterward, the oxygen atomic displacement vector map (distance and direction) is obtained, as shown in Fig. 2d by quantifying the ABF image of Fig. 2b (the calculation process was described in Supplementary Fig. 4), with the yellow arrows indicating the reversed oxygen atom displacement (*D*_O_) vectors. Here, the O_II_ site oxygen columns significantly shift (~13.5% of the c-lattice parameter^23^) from the central position of the four nearest heavy atoms, while the O_I_ site oxygen columns shift slightly (<1% of the c-lattice parameter^23^). The different displacements of the oxygen columns are attributed to their different coordination number, that is, the fourfold O_I_ site oxygen in constant position represents paraelectric behavior, while the shift of threefold O_II_ site oxygen is the origin of ferroelectricity from the microscopic view (see Supplementary Fig. 5). Hence, the visualized map from the above two-dimensional oxygen displacement vectors is illustrated in Fig. 2e. The periodic dark- and light-purple columns on the map fit well with the asymmetric oxygen shift in Pca2_1_ structure. These results visualize the oxygen configuration by using Cs-corrected STEM-ABF technique, which could deeply probe into the intrinsic nature of the ferroelectric o-phase in hafnium/zirconium oxides.

### Switchable Fe diode and 3D integration

**Fig. 3:**
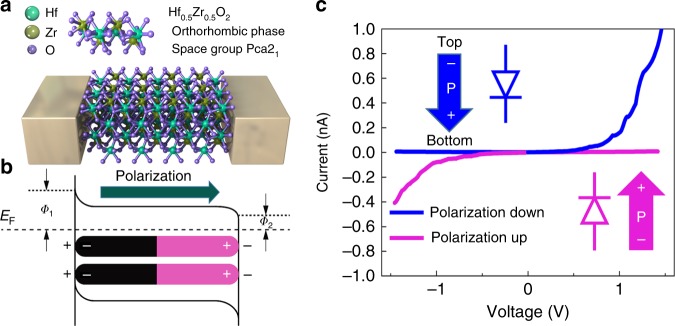
Ferroelectric diode with a thin (∼10-nm) ferroelectric layer. **a** Schematic structure of the ferroelectric diode. **b** Schematics of the energy band diagrams of the Schottky-to-ohmic interfacial contacts in TiN/HZO/TiN modulated by polarization orientations. **c** Nonlinear diode-like *I*–*V* of the TiN/HZO/TiN device with a cell size of 1 μm. Insets represent a schematic of the potential energy profiles in two opposite polarization states.

Based on such a thin (~10-nm) ferroelectric layer (HZO), a switchable Fe-diode device was demonstrated, and the fabrication process is shown in Supplementary Fig. 6. Figure 3a shows the schematic structure of the TiN/HZO/TiN Fe diode, with Schottky contacts formed at the interfaces between the metal electrodes and ferroelectric films. The positive polarization charges can be neutralized by electrons and then induce the accumulation of electrons in the polarization head side, leading to the reduction of the barrier height; the negative polarization charges can be compensated by oxygen vacancies with positive charge, resulting in upward-band bending. Thus, the Schottky-to-ohmic contact was formed in the TiN/HZO/TiN device (Fig. 3b), leading to a diode-like conduction. As the polarization switched, the direction of the diode was changed with the direction of polarization. Figure 3c shows the *I*–*V* characteristics of the Fe diode device (the whole *I*–*V* curve is shown in Supplementary Fig. 7). After sweeping to positive voltage, the current changes from low to high, and the state changes from negative-forward diode to positive-forward diode (blue lines). Similar behavior can be observed in negative voltage sweeping (red lines), where the state changes from positive-forward diode to negative-forward diode. Due to the existence of Schottky barrier between HZO and electrode, this Fe diode exhibits inherent nonlinearity (more than 100), defined as the ratio of the current read at Vr and Vr/2. This attribute is important for suppressing the leakage current in a cross-point array, eliminating the necessity of adding an external selector device in series with each memory cell^24–26^. Read disturbance is a common issue of two-terminal devices, especially the ferroelectric devices due to its accumulative switching of polarization^27^. In the case of polarization state with the same direction of read voltage polarity (e.g., +2 V/100 ns), the read operation has negligible influence on the polarization state (polarization-down, on state) (shown in Supplementary Fig. 8a). However, in the case of polarization state (polarization-down, off state) opposite to the polarity of read voltage, the read pulse will gradually increase the off-state current, leading to disturbance issue. This issue could be solved by a combined pulse read scheme with a pair of positive and negative read pulses. The reverse pulse is helpful to restore the original polarization state. As shown in Supplementary Fig. 8b, by using this method, the off-state current remains almost unchanged after 10^9^ read pulses.

**Fig. 4:**
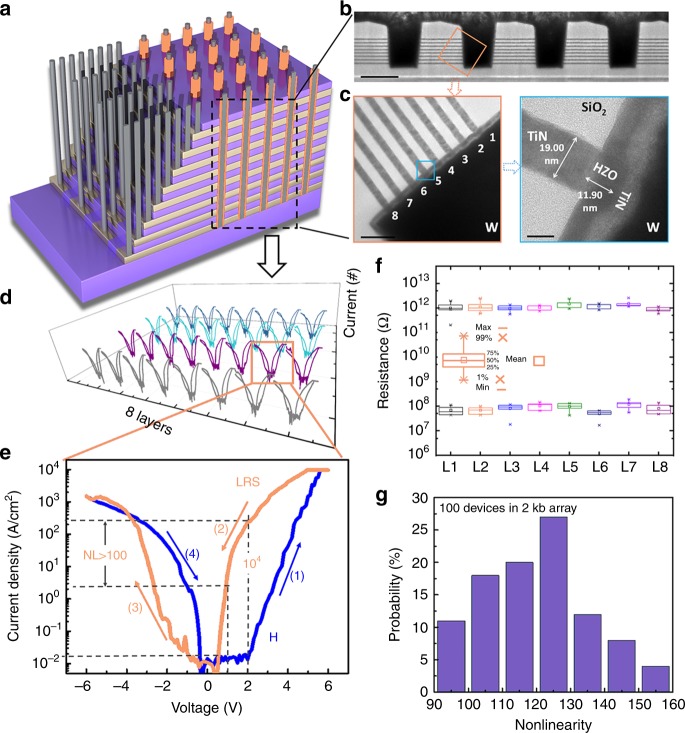
High-density 3D integration of the ferroelectric diode devices. **a** A schematic view of the 8-layer 3D vertical ferroelectric diode (Fe diode) array. **b** Cross section of the 3D vertical structure with Fe diode devices and the detailed structure information for the devices, where a 417.5-nm hole structure and 8-layer vertical memory cells can be observed clearly. Scale bar, 500 nm. **c** The cell size was defined by the thickness of the TiN and the perimeter of the holes (19 nm × 1.31 μm). Scale bar (left), 10 nm. Scale bar (right), 10 nm. **d** *I*–*V* curves of the Fe diode devices in the 4 × 8 array framed in (**a**). **e** Typical *I*–*V* curve of the TiN/Hf_0.5_Z_0.5_rO_2_/TiN/W device. Switchable diode property was achieved. In this device, low operation current (<1 μA) and high nonlinearity (>100) were achieved. **f** Resistance distributions of the switchable diode device in the 8-layer array. **g** Distribution of the nonlinearity in 3D array.

In order to verify the potential of high-density integration, the HZO-based Fe diode was further implemented in a 3D architecture. Figure 4a shows the schematic view of the 8-layer stacked Fe diode array. The detailed process flow is shown in Supplementary Fig. 9. Figure 4b shows the TEM image of the 8-layer vertical structure and the element mapping in the selected zone. The magnified device area is shown in Fig. 4c. TiN/HZO/TiN/W device structure and the interface in between could be clearly observed. Figure 4d shows the *I*–*V* curves of the Fe-diode devices in the 4 × 8 array framed that was given in Fig. 4a. Each cell in the eight-layer vertical structure has similar *I*–*V* curves. The typical *I–V* characteristics of Fe–HfO_2_ diodes are shown in Fig. 4e, with the numerical symbols denoting the sequence of voltage sweeps. This *I*–*V* curve has similar diode-switching behavior as the one shown in Fig. 3c, with forward rectifying (blue curve) occurring due to positive polarization and reversal rectifying (read curve) occurring due to negative polarization. Different memory states could be read out easily by applying a proper read voltage (e.g., 2 V). Large on/off ratio of more than 10^4^ was realized. The current density is as high as ~200 A/cm^2^, which is more than 40 times of the traditional Fe diode^18^. This high current density could make the memory status be detected easily by using the sense amplifiers in the peripheral circuitry. To confirm the high sensing speed, we evaluated the sensing time by using a current-sampling-based sense amplifier (SA) (Supplementary Fig. 10). The simulation was carried out for read low-resistance state (*I*_cell_ = 100 nA) and high resistance state (*I*_cell_ = 5 nA) with 128 cells per BL (Supplementary Figs. 11 and 12). It can be seen that the CSA can sense both high- and low-resistance states within 65 ns, and the margin is larger than 60 mV. By further decreasing the HZO film thickness, lower switching voltages and higher operation current could be obtained (Supplementary Fig. 13). Considering the peripheral circuit design in a memory chip, the sensing current and voltage window should be high enough to guarantee the successful read-and-write operation; comprise should be made by selecting the proper material thickness.

Both HRS and LRS show good nonvolatility and stability, confirmed by the retention test over 50,000 s at 85 °C (shown in Supplementary Fig. 14). To evaluate the array-level performance of the Fe diode device, statistical measurement was carried out on an 8 × 8 × 32 3D array. The corresponding resistance distributions of HRS and LRS reads at 2 V are illustrated in Fig. 4f, showing good uniformity with the notably on/off ratio (~10^4^). By purely using the Schottky emission equation with the barrier height varied with ferroelectric polarization, the theoretical on/off ratio (>10^5^) is larger than the measured one (>10^4^), implying that other conduction mechanisms might be dominant in the OFF state (Supplementary Note 1), such as the trap-assisted tunneling, as found in most of the transition metal oxides^27–31^. Figure 4g displays the distributions of the nonlinearity of 100 cells in the 2-kb array, confirming excellent uniformity in the low-resistance state and outstanding self-selective characteristics. To confirm the large-scale feasibility, read-and-write margins were analyzed based on the device specifications (Supplementary Fig. 15). Owing to its high nonlinearity and on/off ratio, a sufficient read margin (10%) can be obtained for up to 100-kb array in the worst-case (only one BL pulled up and all unselected cells at LRS)^32^ condition. It is not sufficient enough to build large-size array, and further optimization is required to improve the nonlinearity. These aforementioned results suggest that the Fe-diode device has high potential to be used for high-density 3D memory application.

### Ultrahigh speed and high endurance

**Fig. 5:**
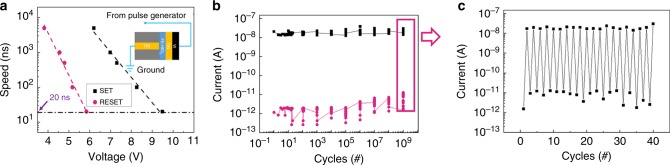
Ultrahigh speed and high endurance. **a** Voltage dependence of the SET and RESET operation speed for Fe-diode memory. The inset shows a schematic of the device structure with the pulse signal. **b** Endurance test. The device can switch >10^9^ pulse cycles. **c** For each order of the cycling number, 20 cycles of write-and-read operation were carried out to confirm the effectiveness of the write pulses.

The switching speed of the Fe diode was measured by using voltage pulse stimulus. We define the forward-diode switching as the SET process and reverse-diode switching as the RESET process. We altered the voltage pulses from a 20-ns to 5-μs width with a magnitude from 3 to 11 V. The ultrahigh operation speed of the Fe diode memory was attributed to the quick polarization flipping of the HZO film, which has been indicated to be able to switch in <20 ns by experimental verification^33^. Figure 5a shows the voltage dependence of the SET and RESET operating speed for Fe diode. Each data point was the mean value of the operation voltage with different pulse widths in Supplementary Fig. 16, showing a compromise between the speed and voltage height. Figure 5b shows the endurance test result under successive voltage pulses with 9.5 V/20 ns for SET and 5.9 V/20 ns for RESET (Supplementary Fig. 17). After 10^9^ cycles, the on-/off-state ratio read by a 2-V voltage pulse can still be well maintained. For each order of the cycling number, 20 cycles of write-and-read operation were carried out to confirm the effectiveness of the write pulses (Fig. 5c). The pulse test results show that this Fe-diode device has robust cycling capability with fast operation speed.

## Discussion

The performance comparison between the HZO-based Fe diode and the perovskite material-based Fe diode is shown in Table 1. Outstanding properties, such as better CMOS compatibility, higher scalability, and higher current density, were achieved in HZO- based Fe diode. Together with its 3D integration capability, the HZO-based Fe diode shows high possibility of combining high speed and high-density features. Thus, complex memory hierarchy composed of working and storage memory is constructed and forms the main building block of the computing system. However, the performance gap between the working and storage memory becomes the bottleneck for system performance improvement, especially in the case of massive data computing, and results in the well-known “memory wall” issue. There is an urgent demand to find a new memory solution with both the merit of high speed and high density. The Fe diode (Supplementary Table 2) demonstrated here shows promising propects on providing unified performance for memory hierachy evolution in the future.

**Table 1:** Comparison of the various reported Fe-diode devices.

Structure	Substrate	Preparation method	Thickness of Fe material	Current density	Nonlinearity	On/off ratio
Au/PbTiO_3_/LSC^16^	LaA1O_3_	Epitaxial deposition	200 nm	0.1 A/cm^2^	2	2
PZT/(LSMO)^38^	SrTiO_3_	PLD	30 nm	0.1 A/cm^2^	2	300
Ag/BFO/Ag^17^	/	/	90 um	20 mA/cm^2^	5	20
Ta/PZT/SRO^39^	SrTiO_3_	PLD	100 nm	2 A/cm^2^	2	1.5
SRO/PZT/SRO^40^	SrTiO_3_	PLD	150 nm	10 mA/cm^2^	2	4
SRO/BFO/Pt^18^	SrTiO_3_	PLD	120 nm	5.4 A/cm^2^	5	4
SRO/BFO/Pt^41^	SrTiO_3_	PLD	40 nm	—	2	753
This work	SiO_2_	ALD	10 nm	200 A/cm^2^	100	10,000

In summary, a 3D-stackable Fe-diode device with high speed and high-density memory characteristics was demonstrated. With the help of polarization reversal, the Fe diode exhibited switchable rectifying behavior. The correlation between the spontaneous polarization and short-range order displacement of the oxygen atom was clearly revealed, which paved a new way to understand the fundamental ferroelectric theory of the hafina-based materials, and figure out effective approaches to optimize the device performance. The demonstration of 3D vertical integration up to 8 layers shows its good potential to achieve high-density storage. Together with the high speed and high-endurance characteristics, this Fe-diode device opens up new opportunities for future memory and storage convergence.

## Methods

### Experimental

(1) Fabrication of planar metal–ferroelectric–metal (MFM) capacitors: Planar MFM capacitors were fabricated on p-doped Si (100) substrates. Initially, TiN bottom electrodes (BEs) of 30-nm thickness were formed by physical vapor deposition (PVD). Thermal ALD at 260 °C was then used to deposit Zr-doped HfO_2_ films from Hf[N(C_2_H_5_)CH_3_]_4_ and Zr[N(C_2_H_5_)CH_3_]_4_. An ALD cycle ratio of 1:1 (Hf-to-Zr precursor pulses) was applied to achieve a Zr content of 50% (cationic ratio of Zr/[Zr + Hf]) in the HfO_2_ layers. Similar to the BEs, 30-nm-thick TiN top electrodes (TEs) were deposited by PVD. The previously amorphous Zr:HfO_2_ films were then crystallized by rapid thermal annealing in an N_2_ ambient at 400 °C for 30 s. The top electron was etched for PFM testing. SiO_2_ thin film was deposited on the HZO film. After optical lithography and etch process, a hole etched down to the HZO film with a diameter of 2 μm was formed. TiN top electron was deposited by ALD; the cell size of Fe diode was defined by the area of contact between TiN and HZO. (2) 3D integration of Fe diode: Preparation of the 8-layer 3D vertical memory with Fe-diode cells: First, multiple TiN (20 nm)/SiO_2_ (30-nm) layers were deposited by PVD and PECVD, respectively. Patterning and only one-step etching were applied to form stacked wordlines (WL) with a smooth sidewall profile. After SiO_2_ filling in the trench, a 500-nm hole is etched down to the bottom SiO_2_. Hf_0.5_Zr_0.5_O_2_ bilayer was deposited on the sidewall sequentially by ALD, followed by depositing of TiN/W by the sputtering to fill the hole as the pillar electrode (BL). Each horizontal WL was opened by selective etching successively. The area of the memory cell was defined by the thickness of the bottom electrode TiN (20 nm) and the perimeter of the hole.

### Characterization

The DC and pulse endurance of a self-selective cell were tested by an Agilent B1500A semiconductor parameter analyzer connected to the experimental device. The pulse measurements were performed using the HV-SPGU module of Agilent B1500A. During the electrical measurement, the W top electrode was biased, while the TiN bottom electrode was grounded.

### STEM part

The annealed TiN/HZO/TiN film was fabricated into a cross-section sample by using focused ion beam (FIB) technique in FEI Helios G4 system, including low-pressure polishing process at 5 and 2 keV, and then cleaning in Gatan 691 PIPS at 1–0.5 keV for removing the residual contamination and possible damage. The scanning transmission electron microscopy (STEM) analysis was carried out on JEM Grand ARM300F microscope operated at 300 kV using STEM mode with probe corrector, including high-angle annular dark-field (HAADF), bright-field (BF), and annular bright-field (ABF) images with a probe convergence semiangle of ~18 mrad. The STEM-HAADF images were taken by an annular dark-field image detector with the inner semiangle larger than 64 mrad. The STEM-ABF images were taken by a BF image detector with the central part being blocked by a beam stopper, in which, the collection semiangle is 12–24 mrad.

### STEM simulation part

We use the software QSTEM^34^ with multislice method to simulate the STEM-ABF and STEM-HAADF images of Pca2_1_ and Pbcm phases; the crystal structures are from CIF files in the literature^34–37^. The thickness of the sample is set to about 20 nm. The microscope parameters are high voltage 300 kV, Cc = 1 mm, convergence angle 18 mrad, defocus 0 mm, spherical aberr. C3 = 0.005 mm, and temperature 300 K. Two detectors are used for the HAADF and ABF, with the inner-to-outer angles 40–200 mrad and 12–24 mrad, respectively. The simulation is along the [010] zone axes, and a series of images tilted from zone axes are also carried out to estimate the effect of tilt to the experimental results.

## Supplementary information

Supplementary Information

## Data Availability

All data needed to evaluate the conclusions are present in the paper and/or the Supplementary Materials. Additional data related to this paper may be requested from the authors.

## Source Data

Source Data
