# Supplementary material for: A highly CMOS compatible hafnia-based ferroelectric diode
Source: Nat Commun. 2020 Mar 13;11:1391. doi: 10.1038/s41467-020-15159-2 (PMC7070068; doi:10.1038/s41467-020-15159-2)
Supplement: Supplementary file 1 — Supplementary Information [file 41467_2020_15159_MOESM1_ESM.pdf]

Supplementary Information

A highly CMOS compatible hafnia-based ferroelectric diode

Qing Luo et al.

Supplementary Figures 1 – 17, Tables 1-2, Note.

Supplementary Figures

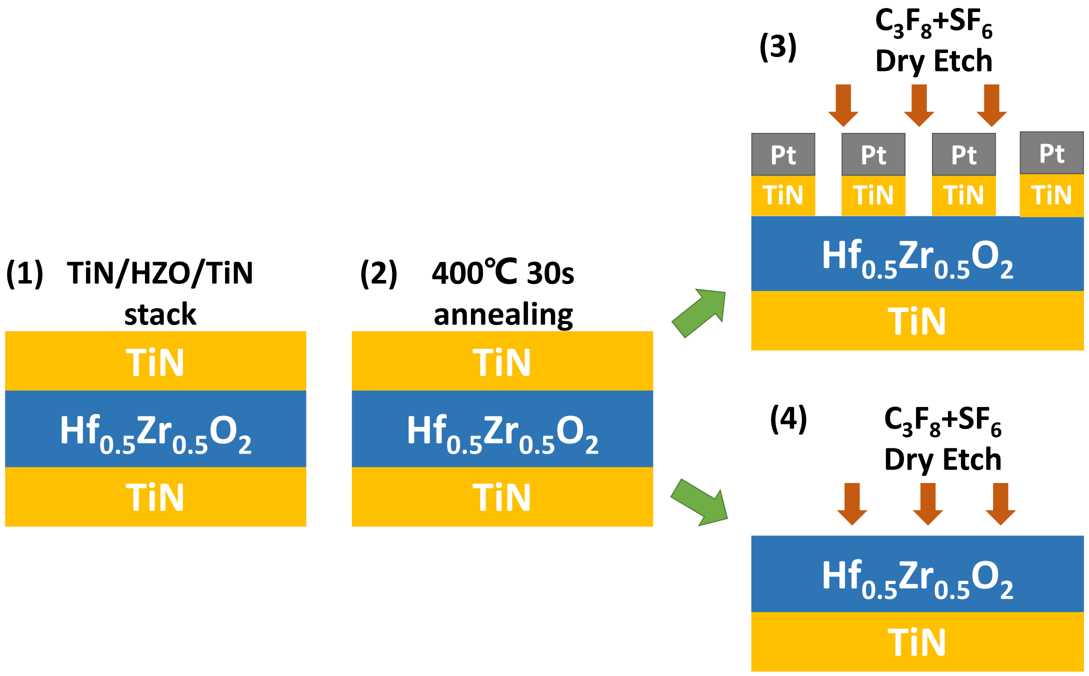

Supplementary Figure 1. The fabrication process of TiN/HZO/TiN device (1) TiN bottom electrodes (BEs) of 30 nm thickness were formed by Physical Vapor Deposition (PVD). An ALD cycle ratio of 1:1 (Hf to Zr precursor pulses) was applied to achieve a Zr content of 50% (cationic ratio of Zr/[Zr + Hf]) in the HZO layers. Similar to the BEs, 30 nm thick TiN top electrodes (TEs) were deposited by PVD; (2) Rapid thermal annealing for crystallization; (3) After annealing, the TiN TE of some samples were patterned by dry etch with Pt as hard mask for P-V loop test and (4) some of them were blank etched to remove the TiN TE for PFM test.

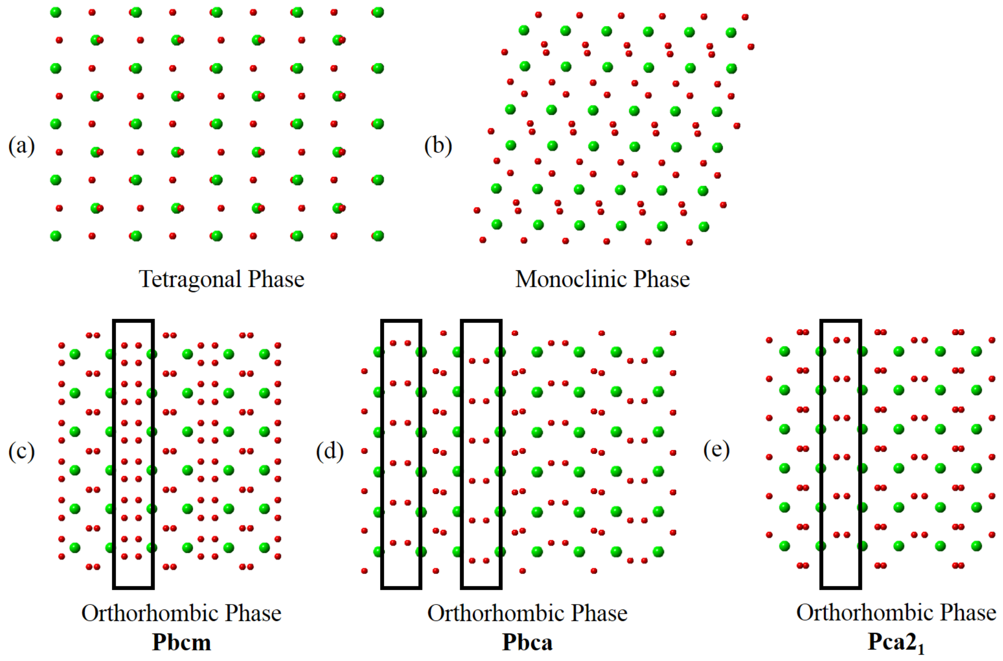

Supplementary Figure 2. Crystal model of four different phase along an axis of [010]. The O-phase can be easily distinguished in (a) T-phase and (b) M-phase; the arrangement of O atoms in the black rectangle is quite different among the O-phase (c) Pbcm, (d) Pbca and (e) $Pca2_1$.

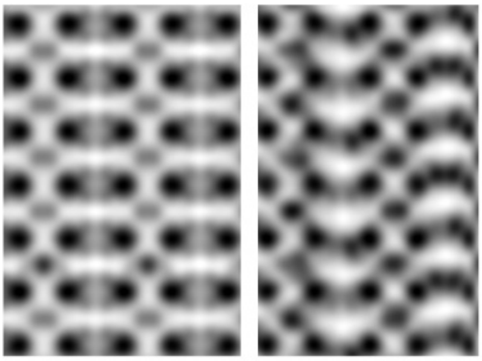

Supplementary Figure 3. Simulated ABF pictures of O-phase with Pbcm and Pbca space groups, which are totally different from experiment result in **Figure 2b**.

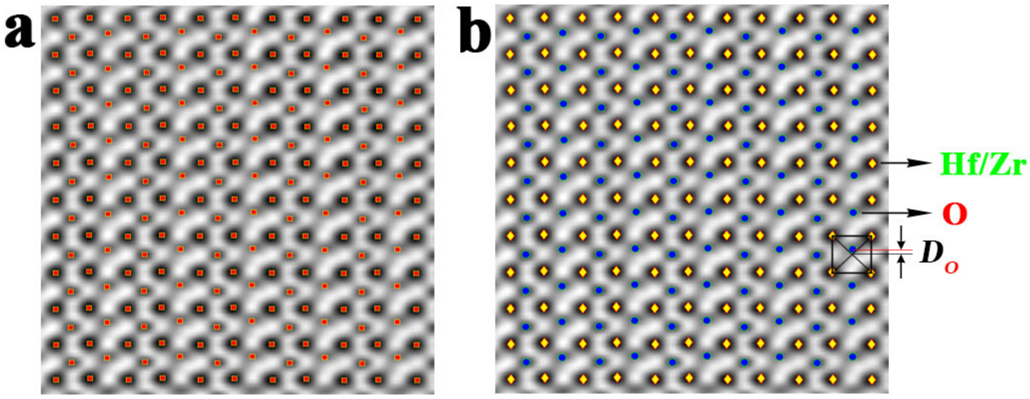

Supplementary Figure 4. The calculation process of O atomic displacement vector. The determination of O atomic columns was confirmed through STEM-ABF image, which was quantified by a reported peaks finding method[1]. Because the two O atomic columns are very close together, we label the two as one when determining their position. Following are the major calculation steps of O atomic displacement vector (distance and direction). First, Gaussian filter for noise reduction was applied to a STEM-ABF image. Second, the O positions were determined accurately by fitting them as 2D Gaussian peaks by using Matlab showing in panel (a). Third, O atomic displacement distance and direction were determined by comparing O atomic columns and the central positions of their corresponding four nearest heavy atoms as shown in panel (b). Hence, according to the data of relative position, we can draw O atomic displacement (D_O) vectors map and distances map by using Matlab.

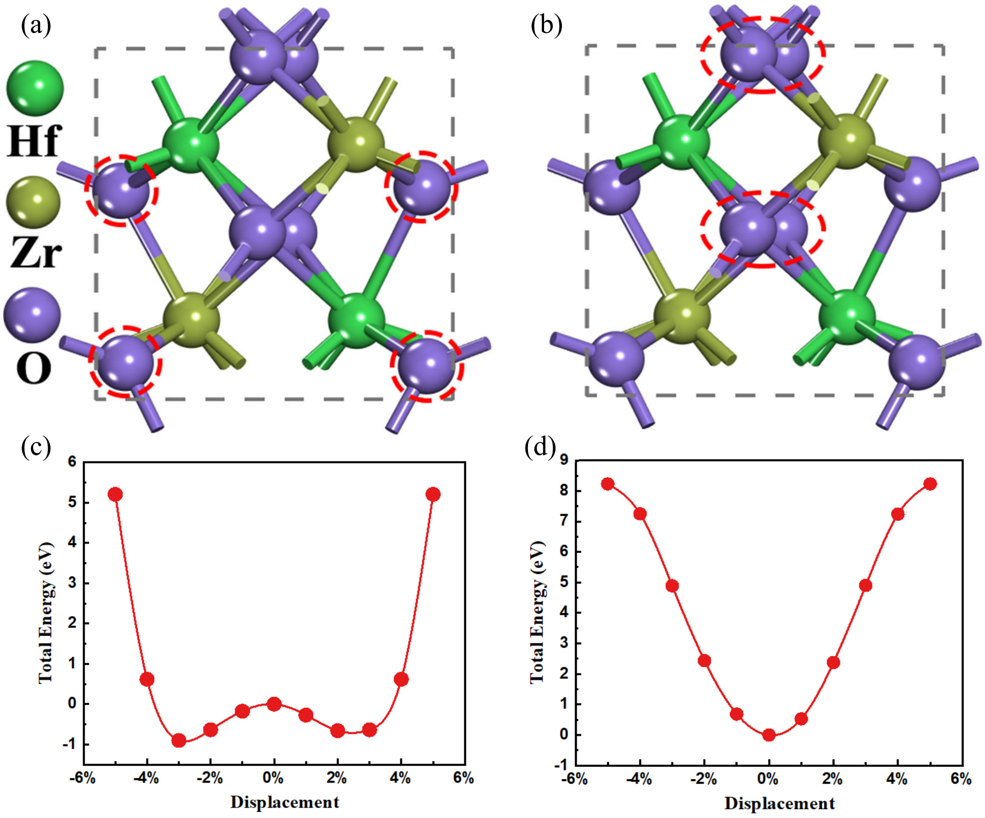

Supplementary Figure 5. First-principles calculations to evaluate the effects of oxygen movement on total energy in $Hf_{0.5}Zr_{0.5}O_2$. Movement of (a) 3-fold coordinated oxygen and (b) 4-fold coordinated oxygen in unit cell of orthorhombic $Hf_{0.5}Zr_{0.5}O_2$. Total energy vs. displacement of (c) 3-fold oxygen and (d) 4-fold oxygen. The total energy is the energy difference relative to 0% displacement.

First-principles calculations based on Density Functional Theory (DFT) are conducted to evaluate the effects of oxygen movement on total energy in $Hf_{0.5}Zr_{0.5}O_2$ using the Quantum ESPRESSO package[2] and thus demonstrate that movement of 3-fold coordinated oxygen is the main reason for polarization. A periodically arranged unit cell (12 atoms) of $Hf_{0.5}Zr_{0.5}O_2$ is obtained by the replacement of half of the Hf atoms with Zr atoms in the orthorhombic-HfO_2 cell ($Pbc2_1$) followed by structural relaxation. **Supplementary Figure 5a, b** show the Movement of (a) 3-fold coordinated

oxygen and (b) 4-fold coordinated oxygen in unit cell of orthorhombic $Hf_{0.5}Zr_{0.5}O_2$. The Perdew-Burke-Ernzerhof (PBE) version of the generalized gradient approximation (GGA) functional[3] is used to optimize the geometric structure and calculate the total energies of a series of structures with displaced 3/4-fold coordinated oxygens. The energy cutoff is set as 60 Ry and k-points as 5×5×5 mesh grid. Ultrasoft pseudopotential is used for all the atoms (Hf, Zr, and O) of $Hf_{0.5}Zr_{0.5}O_2$. **Supplementary Figure5c, d** show the total energy vs. displacement of oxygen. For 3-fold oxygen, the energy shows two stable minima points, which is the origin of ferroelectric distortion. However, for 4-fold oxygen, only one minima point means a paraelectric behavior. Therefore, movement of 3-fold oxygen is the origin of ferroelectricity from microscopic view.

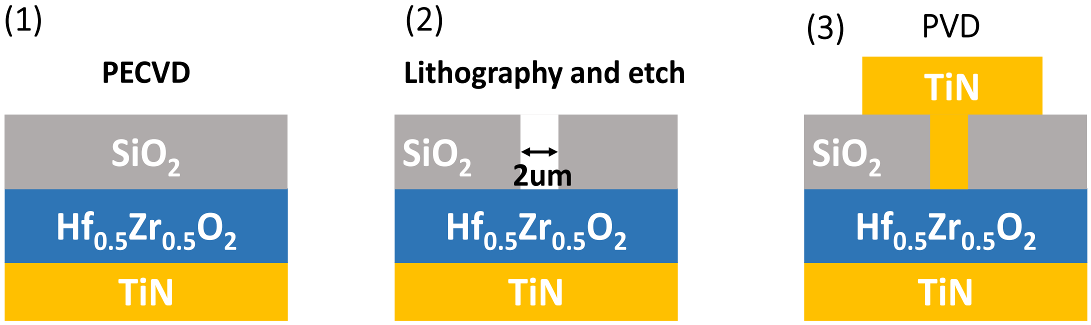

Supplementary Figure 6. Fabrication process planer Fe Diode. (1) Follow the steps in Figure. S1(4). SiO_2 was stacked on the HZO layer by PECVD; (2) Lithography and etching was used to form a hole etched down to HZO film with a diameter of 2 µm; (3) TiN top electron was deposited by ALD, the cell size was defined by the area of contact between TiN and HZO.

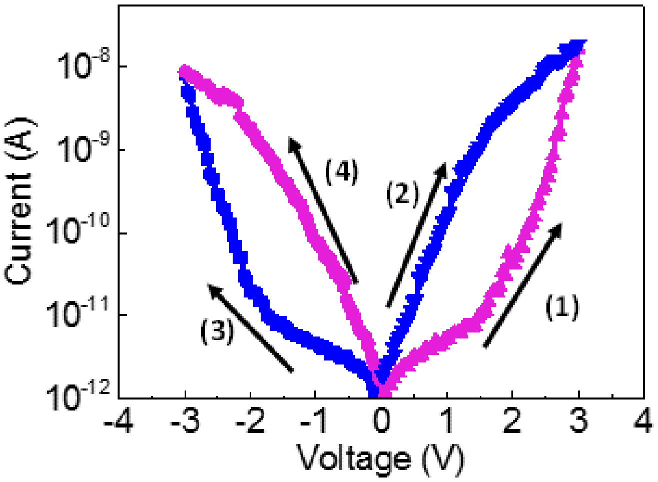

Supplementary Figure 7. Typical IV curves of the Fe-diode.

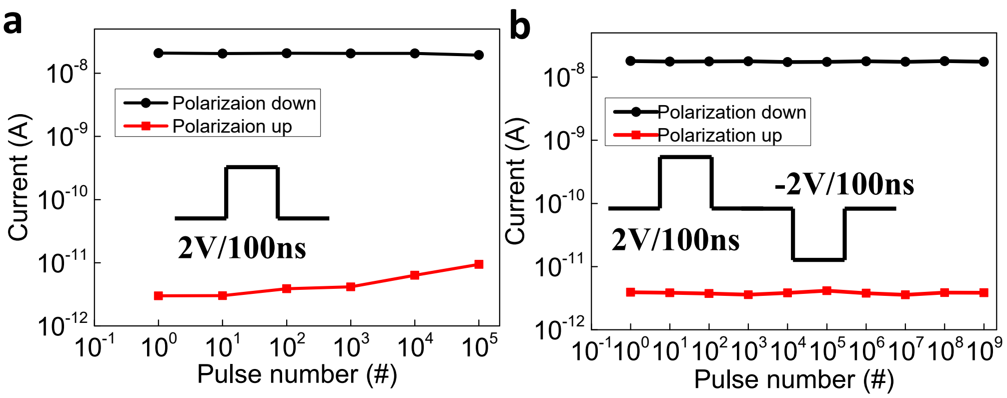

Supplementary Figure 8. Read disturbance (a) for traditional read operation, after 10^5 read pulses off-state current began to increase. (b) for a combined pulse method, the device shows excellent immunity to pulse disturbance.

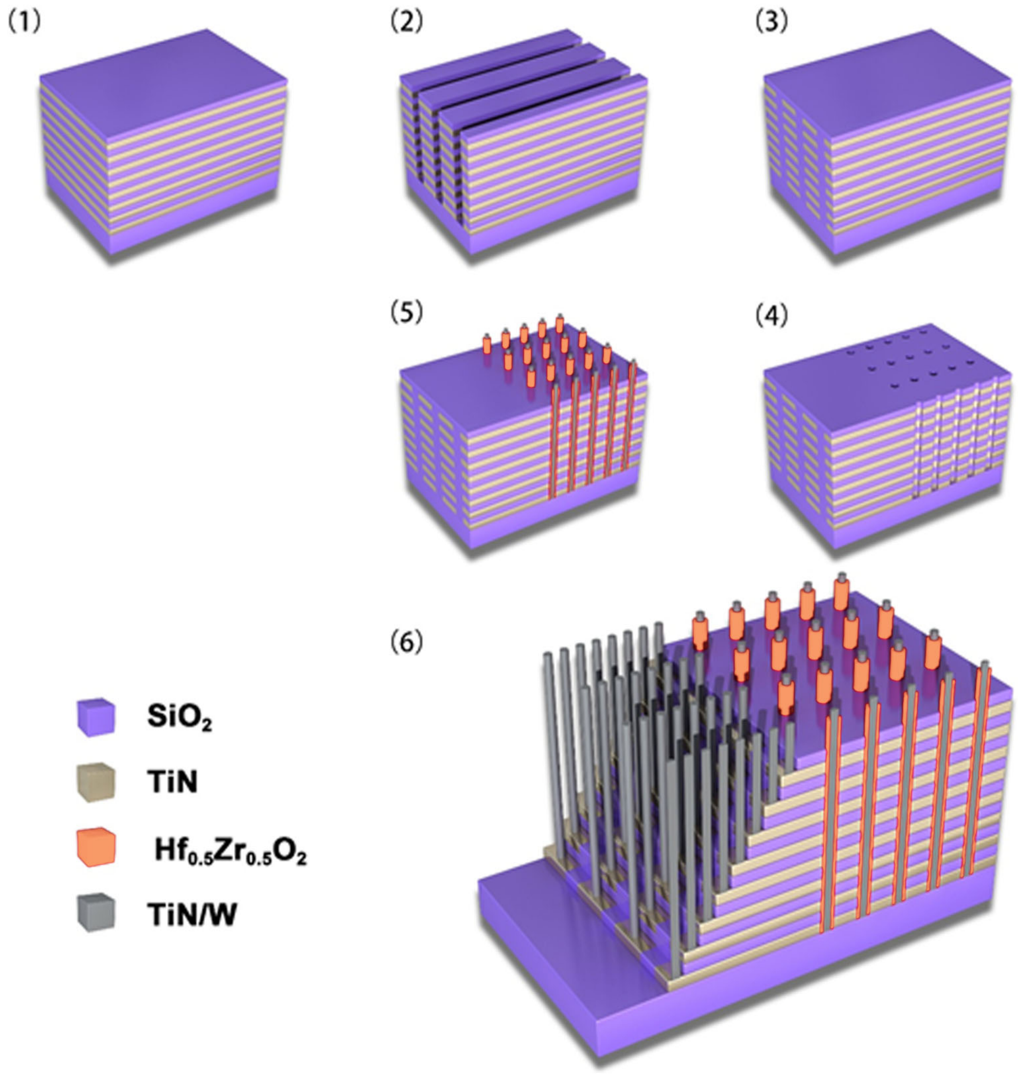

Supplementary Figure 9. Process flow of the 8-layer 3D VRRAM structure: (1) Multiple TiN/SiO_2 are deposited by PVD and PECVD. (2) Dry etch to split the plane electrode. (3) SiO_2 filling in the trench. (4) Hole etching and (5) functional layer

As shown in **Supplementary Figure 9,** multiple TiN (20 nm)/ SiO_2 (30 nm) layers were deposited by PVD and PECVD, respectively. Patterning and staircase etching were applied to form stacked wordlines (WL) with smooth sidewall profile. After SiO_2 filling in the trench, 500nm hole is etched down to the bottom SiO_2. $Hf_{0.5}Zr_{0.5}O_2$ layer

was deposited on the sidewall sequentially by ALD, followed by deposition of TiN/W by the sputtering to fill the hole as the pillar electrode (BL). Each horizontal WL was opened by selective etching successively. The area of the memory cell was defined by the thickness of bottom electrode TiN (20 nm) and and the perimeter of the hole.

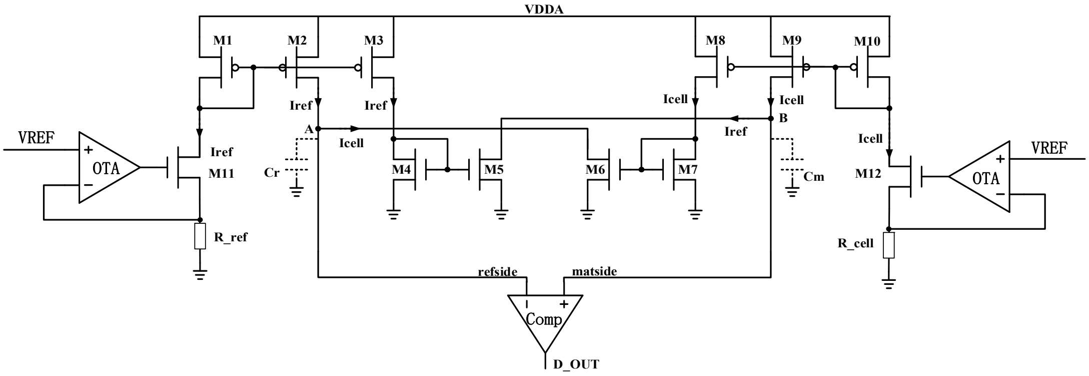

Supplementary Figure 10. Circuitry of current mode sense amplifier.

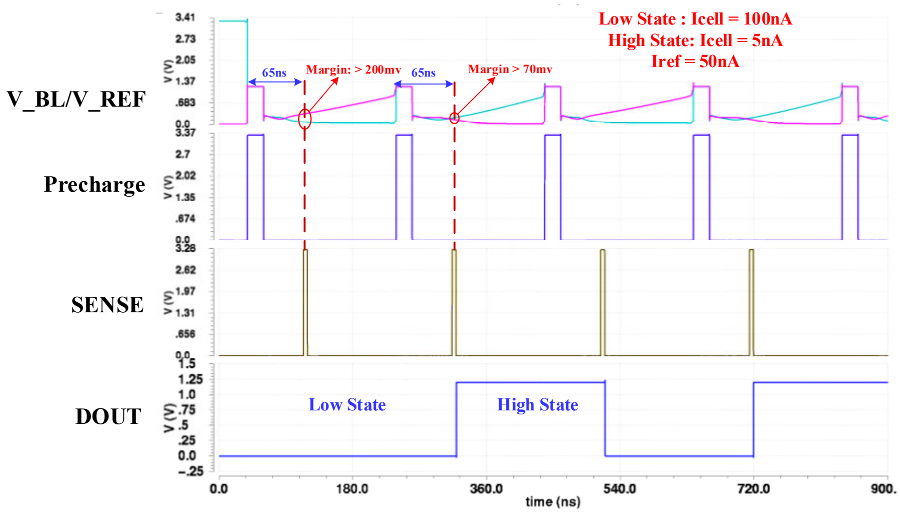

Supplementary Figure 11 Simulation results of CSA. The current of high state is 100 nA and the current of the low state is 5 nA. The reference current is chosen as 50 nA. The BL length is 128 (Cbl = 64 fF) for simulation Bitline loading effect.

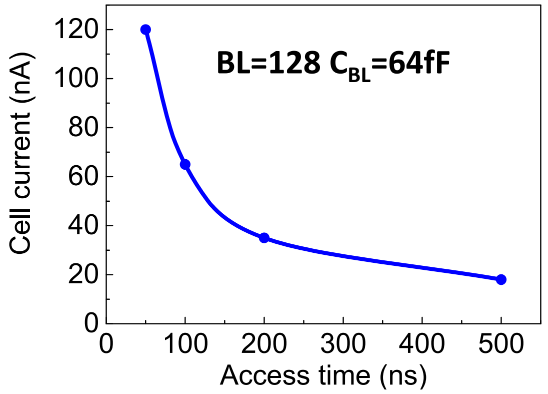

Supplementary Figure 12 Relationship between cell read currents and access speed.

From the circuitry design point of view, the small read current is indeed a big challenge for high speed access. The voltage-mode sense amplifier (VSA) is commonly used in SRAM, PCM, RRAM and MRAM[4-8]. The operating speed of VSA is very sensitive to the resistance values of the memory cell. As the resistance values increases, the operating speed of the VSA drops sharply. In contrast, Current-mode SA (CSA) achieves faster read speeds than VSA for sensing resistor[9,10], its working speed is insensitive to the resistance values of the memory cell, making it suitable for applications with high resistance range. For Fe-diode application, we evaluated the sensing speed by using a current-sampling-based SA, which is capable of detecting sub-100 nA, while providing a read speed within 100 ns.

The sense amplifier consists of three parts as shown in **Supplementary Figure 10**. The first part is a LDO composed of OTA and transistors M1, M10, M11, M12 and resistors R_ref and R_cell. The second part is the current mirror composed of transistors M2 to M9, and the third part is a voltage comparator. The function of the LDO is to convert the resistance of the Fe-diode cell into current, and the function of the current mirror is to convert the obtained current into voltage, and finally the comparator compares the voltage difference to obtain a digital signal. Capacitors Cm and Cr represent the parasitic capacitances associated to the input nodes of the voltage comparator Comp, hereinafter referred to as matside and refside, respectively. The basic working principle is as follows:

The reference current can be expressed as

$$I_{ref}=V_{REF}/R_{\_ref} \qquad (1)$$

and the current of the Fe-diode cell can be expressed as

$$I_{cell}=V_{REF}/R_{\_cell} \quad (2)$$

The current flowing through the transistors M2 to M5 is the reference current I_{ref}, and the current flowing through the transistors M6 to M9 is the current I_{cell}. According to Kirchhoff's current law, the currents flowing to the two input parasitic capacitances C_r and C_m of the comparator are

$$I_{ref}-I_{cell} \quad (3)$$

and

$$I_{cell}-I_{ref} \quad (4)$$

respectively. When the resistance value $R_{\_cell}$ of the Fe-diode is smaller than the reference resistance $R_{\_ref}$, the current I_{cell} flowing through the Fe-diode cell is greater than the reference current I_{ref}, the parasitic capacitance C_r is discharged on the comparator refside, the voltage at the point A is lowered, and the parasitic capacitance C_m is charged on the matside of the comparator. The voltage at point B rises and the comparator outputs a high level "1". On the contrary, when the resistance value $R_{\_cell}$ of the Fe-diode cell is greater than the reference resistance $R_{\_ref}$, the current I_{cell} flowing through the Fe-diode is smaller than the reference current I_{ref}, the parasitic capacitance Cr is charged on the comparator refside, the voltage at the point A is increased, and the parasitic capacitance of the comparator matside is opposite. As a result, Cm is discharged, and the voltage at point B decreases, with the comparator outputting a low level “0”.

Thanks to the symmetric structure, the CSA has tolerance for process variation and

residual current, which reduces the time required for BL precharge. Compared to other SAs, the CSA achieves faster read speeds.

Supplementary Figure 11 plots the simulation results of CSA for read low resistance state (I_{cell} = 100 nA) and high resistance state (I_{cell} = 5 nA) with 128 cells per BL. It can be seen that the CSA can sense both high resistance state and low resistance state within 65 ns, and the margin is larger than 60 mv.

In order to compare the effect of different cell currents on the read speed of CSA, we simulated the read speed covering the read current range from 50 nA to 500 nA, as shown in **Supplementary Figure** 12. It can be seen that the CSA can get the sensing speed within 120 ns at 50 nA. When the read current of the memory cell reaches 200 nA, the access speed of the circuit reaches 35 ns, and when the current of the cell is increased to 500nA, the speed of the circuit is as high as 18 ns.

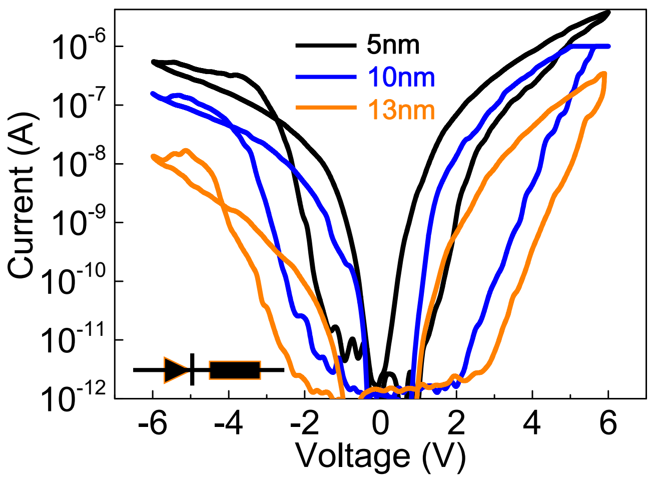

Supplementary Figure 13. I-V curves of HZO device with different thicknesses.

Note: 10 nm device shows the property with high on-off ratio, high nonlineairty, while the thinner films (i.e. 5nm) exhibit higher current density and lower swtiching voltages. The inset show the equivalent circuit diagram of the Fe-diode. The interface effect is equivalent to a diode and the bulk resistance is equivalent to a resistor. By further decreasing the HZO film thickness, lower switching voltages and higher operation current could be obtained due to the decreasing of the bulk resistance.

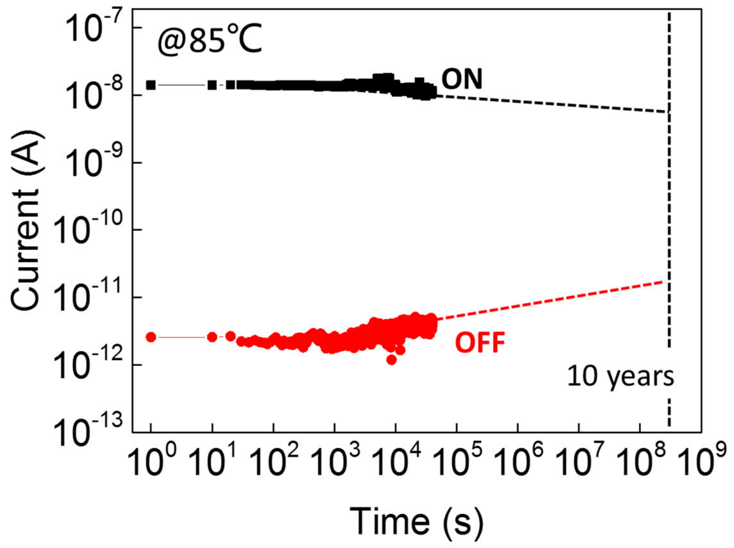

Supplementary Figure 14. The retention of the device for more than 50000s @85℃

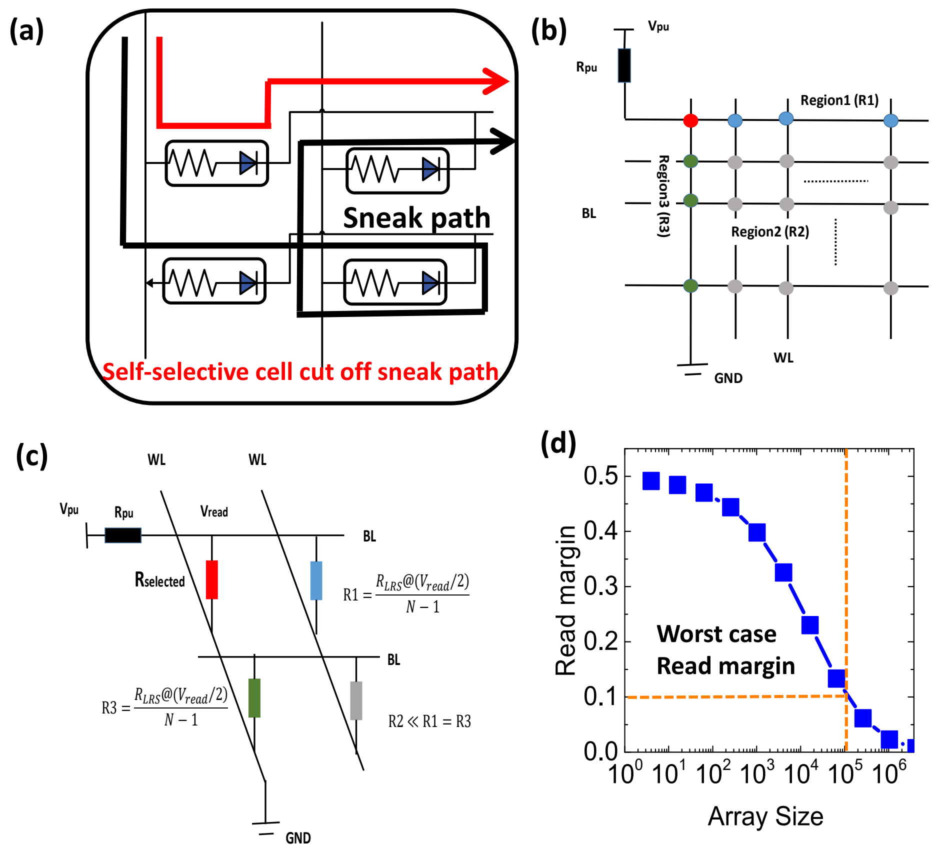

Supplementary Figure 15. (a) A schematic diagram of the typical crossbar array showing the read disturbance problem by the presence of sneak current: read current (red line), sneak current (black line). The sneak current through the on-state neighboring memory cells disturbs the reading out of the off-state cell. In the worst case scenario, all the neighboring memory cells are in on-state, making the problem even more serious; b) Schematic of a square crossbar array. Unselected cells can be divided into three regions: R1, R2 and R3; c) The equivalent circuit in the worse-case scenario (only one BL pulled up and all unselected bits at LRS). Vread is dropped on the selected cell, while Vread/2 is dropped on the cells in the same row or column of selected cell. d) A sufficient read margin (10%) can be obtained up to 100 kb array in the worst case condition.

Crossbar can ideally offer the smallest footprint of cell size - $4F^2$ (F is the feature size)[11]. However, the crossbar structure has a serious problem called the sneaking current issue[12]. Taking a 2×2 array as an example, if the three neighbor cells are at low-resistance state (LRS), whatever the state of designated cell is, its readout state will always be LRS, resulting in reading error or cross talk (**Supplementary Figure 15a**). The larger array size is, the more serious cross talk will be. To inhibit the sneaking current from the unselected cells, high nonlinearity is necessary for the memory cell, by integrating separate selector device as a common solution.[13].

The Fe-diode demonstrated here exhibits spontaneous high nonlinearity (more than 100) resulted from the exponential current-voltage relation of Schottky contact, allowing built-in selector functionality. **Supplementary Figure 15b** shows the worst case (only one BL pulled up and all unselected cells at LRS)[14] of reading with only one BL pulled up and all unselected cells are at LRS. The equivalent circuit for calculating sense margin could be found in the **Supplementary Figure 15c**. Owing to its high nonlinearity and on/off ratio, a sufficient read margin (10%) can be obtained up to 100 Kb array in the worst case condition, as shown in **Supplementary Figure 15d.**

The quantitative assessment on the read margin △V normalized to the pull-up voltage Vpu can be calculated by solving the Kirchhoff equation:

$$\frac{\Delta V}{V_{pu}} = \frac{R_{pu}}{R_{LRS}(V_{read}) \left\| \frac{2R_{LRS}(\frac{V_{read}}{2})}{N-1} + R_{pu}\right.} - \frac{R_{pu}}{R_{HRS}(V_{read}) \left\| \frac{2R_{LRS}(\frac{V_{read}}{2})}{N-1} + R_{pu}\right.} \quad (5)$$

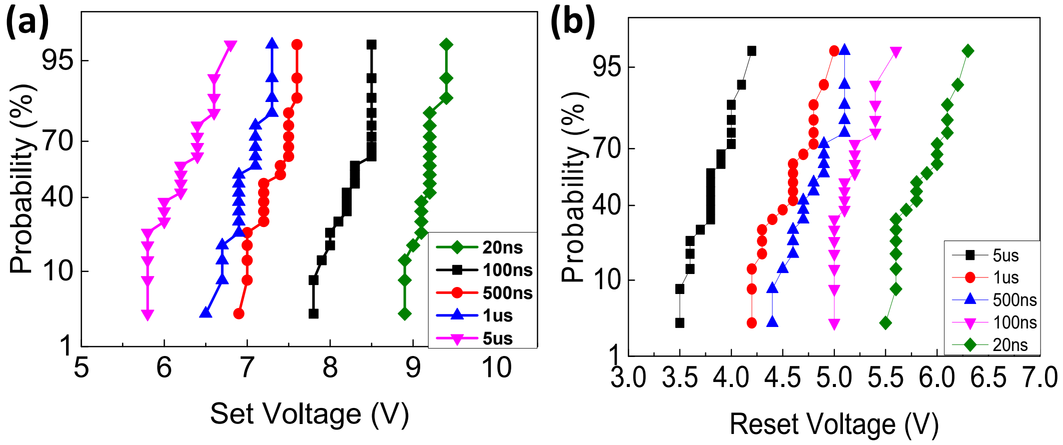

Supplementary Figure 16. Set and reset pulse amplitude cumulative probability for pulse durations ranging from 5 µs down to 100 ns.

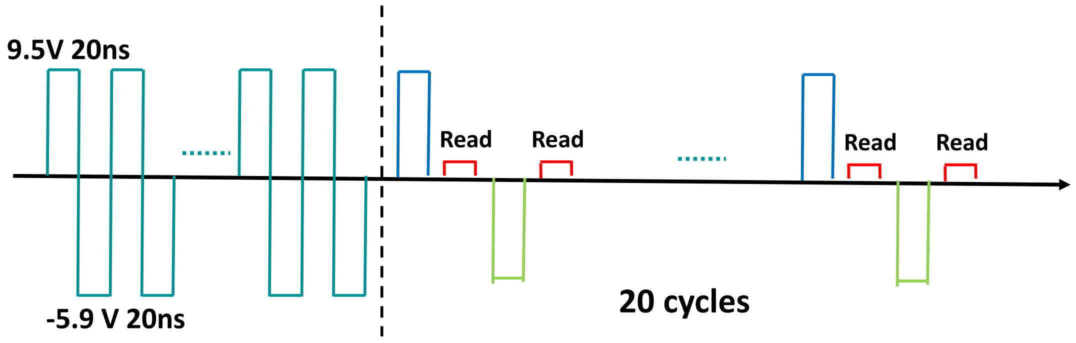

Supplementary Figure 17. Schematic of the pulse test in Fig. 5d. For each order of during the cycling number, 20 cycles write and read operation was carried out to confirm the effectiveness of the write pulses.

Supplementary Table

Structure	Fe-memory	Non-distructive readout	3D stackable	Random access in 3D structure
1T1C	FRAM	No	No	Yes
1T	Fe-FET	Yes	Yes (vertical)	No
1R	FTJ	Yes	No (additional selection device is required)	/
	Fe-diode	Yes	Yes (vertical, built-in nonlinearity)	Yes

Supplementary Table 1. Comparison between 1T/1C, FeFET, FTJ and Fe-diode device

	DRAM	3D NAND	HZO-based Fe-diode
Structure	1T1C	1T	1R
3D-stackable	No	Yes	Yes
Write speed	~10 ns	1 µs	20 ns
Endurance	10^{15}	10^{5}	10^{9}
Retention	~ms	10 years	10 years

Supplementary Table 2 Comparison of the main properties of DRAM, 3D NAND and HZO-based Fe diode.

Supplementary Notes

Supplementary Note 1: According to the model of metal-ferroelectric interface[15, 16], the variation in the built-in potential with ferroelectric polarization can be given by

$$\Delta\Phi_{bi} = \Phi'_{bi} - \Phi_{bi} = \pm P\delta/\varepsilon_0\varepsilon_s \quad (1)$$

, where Φ_{bi} is the built-in potential without contribution from polarization, Φ'_{bi} is the built-in potential with contribution from polarization, the apparent $\varepsilon_0$is the permittivity of free space, ε_s is the static dielectric constant, P is ferroelectric polarization, and δ is the thickness of a interface layer between the surface polarization charge and the metallic electrode. The value of δ is estimated to be the order of a unit cell[17]. For this work the remanent polarization is ~17 uC/cm^2, and dielectric constant is about ~30[18]. Then the variation in the built-in potential $\Delta\Phi_{bi}$ can be as high as 0.32V for δ value of 0.5 nm. Considering that the Schottky emission equations is:

$$J = A^{*}T^{2}\exp\left[\frac{-q(\phi_B - \sqrt{qE/4\pi\varepsilon_0\varepsilon_r})}{kT}\right] \quad (2)$$

By purely using the Schottky emission equation, the theoretical on/off ratio is about exp (q$\Delta\Phi_{bi}$/kT)=exp(0.32/0.025)=3.6×10^5, which is larger than the measured on/off ratio (>10^4). This result implies that other conduction mechanism may be dominant in OFF state, such as trap-assisted-tunneling, that is shown in majority of transition metal oxides[19, 20].

Supplementary References

1 Yin, W., Huang, R., Qi, R. & Duan, C. Extraction of structural and chemical information from high angle annular dark-field image by an improved peaks finding method. *Microsc. Res. Techniq.* **79**, 820-826 (2016).

2 Giannozzi, P. *et al.* QUANTUM ESPRESSO: a modular and open-source software project for quantum simulations of materials. *J Phys. Condens. Matter.* **21**, 395502 (2009).

3 Perdew, J. P., Burke, K. & Ernzerhof, M. Generalized gradient approximation made simple. *Phys. Rev. Lett.* **77**, 3865-3868 (1996).

4 Takeuchi, K. *et al.* A 56-nm CMOS 99 mm_2 8-Gb Multi-Level NAND Flash Memory With 10-MB/s Program Throughput. *IEEE J. Solid-St. Circ.* **42**, 219-232 (2006).

5 Byeon, D.-S. *et al.* An 8 Gb multi-level NAND flash memory with 63 nm STI CMOS process technology in *2005 IEEE International Digest of Technical Papers. Solid-State Circuits Conference(ISSCC),* 46-47 (2005).

6 Chang, S.-H. *et al.* A 48nm 32Gb 8-level NAND flash memory with 5.5 MB/s program throughput. in *2009 IEEE International Solid-State Circuits Conference (ISSCC).* 240-241 (2009).

7 Marotta, G. *et al.* A 3bit/cell 32Gb NAND flash memory at 34nm with 6MB/s program throughput and with dynamic 2b/cell blocks configuration mode for a program throughput increase up to 13MB/s. in *2010 IEEE International Solid-State Circuits Conference (ISSCC).* 444-445 (2010).

8 Calhoun, B. H. & Chandrakasan, A. A 256kb sub-threshold SRAM in 65nm CMOS. in *2006 IEEE International Solid State Circuits Conference (ISSCC).* 2592-2601 (2006).

9 Chang, M.-F. *et al.* A 0.5 V 4Mb logic-process compatible embedded resistive RAM (ReRAM) in 65nm CMOS using low-voltage current-mode sensing scheme with 45ns random read time. in *2012 IEEE International Solid-State Circuits Conference (ISSCC).* 434-436 (2012).

10 Wei, L. *et al.* A 7Mb STT-MRAM in 22FFL FinFET technology with 4ns read sensing time at 0.9 V using write-verify-write scheme and offset-cancellation sensing technique. in *2019 IEEE International Solid-State Circuits Conference (ISSCC).* 214-216 (2019).

11 Goodwin, W. E. P. (Google Patents, 1954).

12 Huang, J.-J., Tseng, Y.-M., Luo, W.-C., Hsu, C.-W. & Hou, T.-H. One selector-one resistor (1S1R) crossbar array for high-density flexible memory applications. in *2011 IEEE International Electron Devices Meeting (IEDM),* 31.37. 31-31.37. 34 (2011).

13 Seok, J. Y. *et al.* A review of three-dimensional resistive switching cross-bar array memories from the integration and materials property points of view. *Adv. Funct. Mater.***24**, 5316-5339 (2014).

14 Yoon, K. J., Bae, W., Jeong, D. K. & Hwang, C. S. Comprehensive writing margin analysis and its application to stacked one diode-one memory device for high-density crossbar resistance switching random access memory. *Adv. Electron. Mater.* **2**, 1600326 (2016).

15 Pintilie, L. & Alexe, M. Metal-ferroelectric-metal heterostructures with Schottky contacts. I. Influence of the ferroelectric properties. *J. Appl. phys.* **98**, 124103 (2005).

16 Wang, C. *et al.* Switchable diode effect and ferroelectric resistive switching in epitaxial $BiFeO_3$ thin films. *Appl. Phys. Lett.* **98**, 192901 (2011).

17 Scott, J. F. *Ferroelectric memories*. Vol. 3 (Springer Science & Business Media, 2000).

18 Park, M. H. *et al.* Ferroelectricity and antiferroelectricity of doped thin HfO_2 based films. *Adv. Mater.* **27**, 1811-1831 (2015).

19 Higashi, Y. *et al.* New insights into the imprint effect in FE-HfO_2 and its recovery. in *2019 IEEE International Reliability Physics Symposium (IRPS).* 1-7 (2019).

20 Florent, K. *et al.* Investigation of the endurance of FE-HfO 2 devices by means of TDDB studies. in *2018 IEEE International Reliability Physics Symposium (IRPS).* 6D. 3-1-6D. 3-7 (2018).
